# Alien Rainbow Trout *Oncorhynchus mykiss* in the Balkhash Basin (Kazakhstan, Central Asia): 50 Years of Naturalization

**DOI:** 10.3390/ani14203013

**Published:** 2024-10-18

**Authors:** Nadir Shamilevich Mamilov, Marlen Tursynali, Gulnur Kuanyshkyzy Khassengaziyeva, Jan Urban, Dinara Bartunek, Sayat Ermukhanbetovich Sharakhmetov, Nazym Sapargaliyeva, Zhansulu Urgenishbayeva, Gulnar Bolatovna Kegenova, Eleonora Kozhabaeva, Mirgaliy Baimukanov, Boris Levin

**Affiliations:** 1 Department of Biodiversity and Bioresources, Faculty of Biology and Biotechnology, Al-Farabi Kazakh National University, Al-Farabi av., 71, Almaty 050040, Kazakhstan; perca.kessler@gmail.com (M.T.); g96-17@mail.ru (G.K.K.); sharakhmetov@gmail.com (S.E.S.); sapargalyeva.nazym@gmail.com (N.S.); urgenishbaevazh@gmail.com (Z.U.); gkegenova78@gmail.com (G.B.K.); e.b.kozhabaeva@gmail.com (E.K.); 2 Laboratory of Signal and Image Processing, Institute of Complex Systems, Faculty of Fisheries and Protection of Waters, CENAKVA, University of South Bohemia in Ceske Budejovice, Zámek 136, 373 33 Nové Hrady, Czech Republic; urbanj@frov.jcu.cz (J.U.); d.bekkozhayeva@gmail.com (D.B.); 3 Institute of Hydrobiology and Ecology, Irgeli Village, Rakhmetov Str., 58/1, Almaty 040916, Kazakhstan; institute_he@ihe.kz; 4 Papanin Institute for Biology of Inland Waters, Russian Academy of Sciences, Yaroslavl Prov., 152742 Borok, Russia; borislyovin@gmail.com; 5 A.N. Severtsov Institute of Ecology and Evolution of the Russian Academy of Sciences, Leninsky pr., 33, 119071 Moscow, Russia

**Keywords:** fish introduction, grow, diet, population, competition, thermal tolerance, skin hue, life history

## Abstract

**Simple Summary:**

The current state of rainbow trout, *Oncorhynchus mykiss*, was investigated 50 years after the first introduction of the species to the water bodies of the Balkhash basin. The wild form of the species from Kamchatka and the cultured form from European fish farms have successfully adapted to the local environmental conditions. Unlike in most other regions of the world, rainbow trout have not spread beyond the water bodies of their original introduction and have not significantly impacted native fish diversity.

**Abstract:**

Rainbow trout, or mykiss (*Oncorhynchus mykiss*), is one of the most popular species used in aquaculture and has been naturalized worldwide, including in the Central Asian Balkhash basin, which has unique aboriginal fish fauna. Both rainbow trout from European farms and wild mykiss from Kamchatka were introduced to some mountain lakes and rivers of the Balkhash basin about 50 years ago. This study investigates the current distribution and life history traits of the alien species and its possible impact on the local fish fauna. This study showed that the rainbow trout occupies various habitats in the Ili River basin: mountain lakes, fast-flowing mountain rivers, and lowland rivers with slow currents and warm water (up to +27 °C). Rainbow trout from European fish farms dominate the mountain Middle Kolsay Lake, while the wild trout from Kamchatka occupies the small Ulken Kokpak River. Both co-occur in the Chilik River. Contrary to that in other regions, the distribution of rainbow trout in the Balkhash basin remained almost the same after their introduction. Broad intrapopulation variability in terms of size, growth rate, and maturation age was revealed, apparently as a result of adaptation to the new environment and intrapopulation competition. In particular, the growth rate has decreased, but life span, surprisingly, has increased as compared to the originally introduced fish. Intrapopulation variation in growth and maturity patterns was also noted. Differences in skin coloration between highland (cold-water) and lowland (warm-water) populations were discovered. The feeding mode of naturalized trout is insectivorous (insect imago), indicating that it occupies its own niche in the local fish communities. The largest population of rainbow trout was recorded in the Lower Kolsay Lake, lowering the population of native fish species, while in other localities, no negative impact on local fish communities was recorded.

## 1. Introduction

*Oncorhynchus mykiss* (Walbaum, 1792)—commonly called the rainbow trout, steelhead trout, and redband trout—has long been one of the most popular and important species in terms of aquaculture [1]. The native area of the species includes cold-water tributaries of the Pacific Ocean in Northeast Asia and Northwest America [2,3,4]. Fertilized eggs of the rainbow trout were first imported to Germany from California in 1882, and during the last century, the species was distributed on all continents except for Antarctica [5,6]. Currently, naturalized rainbow trout is considered one of the most aggressive and dangerous alien species threatening indigenous aquatic fauna [6,7,8,9].

**Figure 1:**
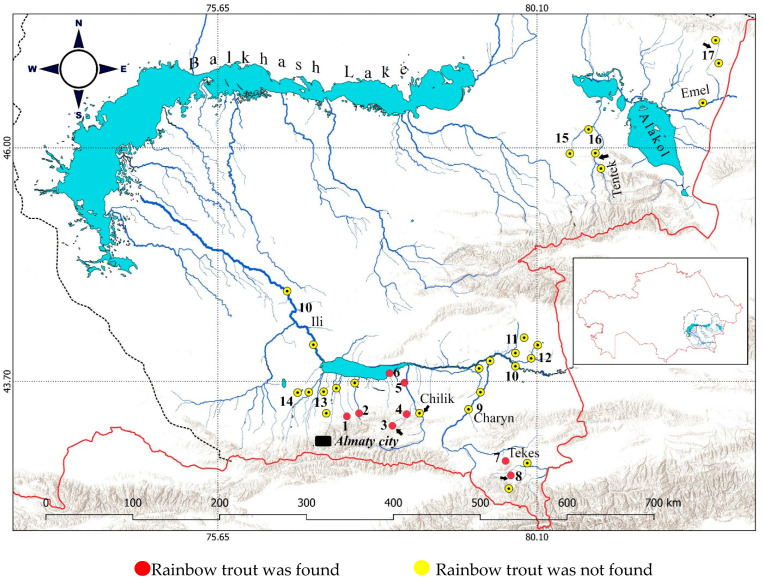
Schematic map of the Balkhash basin. Study sites are indicated by numbers: 1, Issyk River; 2, Turgen River; 3, Kolsay and Kaindy Lakes; 4, Zhinishke River; 5, Chilik River branches near mouth (Masak and Babatogan); 6, Kapchagay Reservoir; 7, Shalkudysu River; 8, Ulken Kokpak River; 9, Charyn River; 10, Ili River; 11, Borokhudzir River; 12, Usek River; 13, Bolshaya Almatinka and Malaya Almatinka Rivers; 14, Kaskelen River; 15, Shynzhyly River; 16, Tentek River (upper and middle sections); 17, Emel River. Arrows show the location of first release of the rainbow trout in the 20th century.

The native ichthyofauna of the Balkhash basin consists of 10–12 species, including 6 that are endemic [10,11,12]. Therefore, in the mid-20th century, the water bodies of the basin were considered by some ichthyologists to be an “ecological vacuum” that had to be filled in order to increase fish production [13]. For this purpose, more than 20 fish species were introduced to the Balkhash basin in the second half of 20th century, including the rainbow trout [14,15,16]. Farmed rainbow trout from Western Europe were introduced into the alpine lakes of Lower Kolsay and Middle Kolsay and the mountainous sector of the Chilik River between 1964 and 1969 [15]. Later, wild rainbow trout from the rivers of the Kamchatka Peninsula were released into high-mountain inflows of the Tekes and Chilik Rivers within the Ili River watershed as well as to the Tentek and Emel River in the Alakol Lake basin [16]. Sites of the first introductions are presented in Figure 1, and the number of introduced fish is given in a Supplementary Table (Table S1). Shortly after their introduction, the rainbow trout became numerous (i.e., naturalized) in the Lower and Middle Kolsay Lakes and in the whole Chilik River [15]. The success of the first introductions provoked the further introduction of the rainbow trout from various European farms to mountain water bodies of other areas in Central Asia, including Uzbekistan and Kyrgyzstan [17,18].

The first data relating to the biology and morphology of the rainbow trout in Kolsay Lakes were comprehensively documented over 25 years following their introduction [15]. In recent years, the population size of the rainbow trout in Kolsay Lakes has been assessed as being dynamically balanced [19,20]. The rainbow trout were found in the Chilik, Turgen, and Issyk rivers [21,22]. Nothing is known about the naturalization of the rainbow trout from Kamchatka. At the same time, issues concerning the current distribution, biology, morphology, and ecology of the introduced populations of the rainbow trout in the Balkhash basin as well as the possible impact on the indigenous fish fauna are largely unstudied. Generally, *O. mykiss* is very plastic in terms of its life history, and the species is characterized by two main life strategies—resident (referred to as rainbow trout) and anadromous (steelhead)—with several intermediate forms between them [23,24,25,26,27,28].

The aims of our study were as follows: (1) to clarify the current distribution of the rainbow trout in the Balkhash basin and life history trait variations of populations, and (2) to assess the possible impact of the naturalized rainbow trout on local fish fauna.

## 2. Materials and Methods

### 2.1. Study Area and Sampling

The field study was conducted from 2020 to 2023. Each water body was sampled at least two times (summer and autumn). The geographical coordinates, brief designations of sampling sites, and number of individuals studied are given in Table S2. Among the sites, Kolsay Lakes, Lake Kaindy, Zhinishke River, and Shalkudysu River are connected to Charyn River. The Upper Kolsay Lake, Middle Kolsay Lake, and Lower Kolsay Lake are located on the Kolsay River, which is a tributary of the Chilik River. The Masak system of springs and brooks and Babatogan channels belong to the lower section of the river. The Ulken Kokpak is a tributary of the Tekes River. The Tekes, Chilik, Charyn, Borokhudzir, Usek, Turgen, Issyk, Kaskelen, and Malaya Almatinka (with inflow of the Bolshaya Almatinka River) rivers connect to the Ili River. Rainbow trout fish farms can be found on the Turgen, Issyk, and Kaskelen rivers. The Tentek and Emel rivers belong to the Alakol Lakes system (Figure 1).

Fishing was carried out using sein nets in rivers and brooks, (20, 5, and 1 m in length, each with a 1.2 m height and a mesh size of 5 mm), gill nets (mesh sizes of 20, 30, 40, 50, and 60 mm, each with a length of 25 m and a depth of 2 m, working overnight) in the Lower Kolsay Lake and Kapchagay Reservoir, and by hook-and-line angling in all water bodies, as permitted by relevant the Almaty oblast and Balkhash-Alakol authorities and Programme of Nature Monitoring of the Alakol State Reserve and the “Kolsay Koldery” National Nature Park. In total, 10 to 30 km of each river was inspected. Each appropriate site in the rivers was fished for about 40 min per visit. In addition, the fisherman’s catch was inspected.

Water quality measurements were performed simultaneously with rainbow trout sampling. The temperature, pH, salinity, and turbidity were measured. Water turbidity was determined using a HI97303 turbidity meter; salinity, temperature, and pH were measured using an HI98129 device (all from Hanna Instruments, Cluj Napoka, Transilvania).

### 2.2. Fish Treatment

Fish measurements were carried out according to recommendations provided in previous studies [29,30]. The total length (TL) and standard length (SL) were measured using calipers with a precision of ±1 mm, and weight was measured (Q) with a precision of ±0.01 g. Fork length (FL) was used to compare the growth rate with the data obtained by Sidorova [15] and Biryukov [16] from the first fish introduced from Europe and Kamchatka, respectively (see Figure S1). The quantity of mesenteric fat and sexual maturity were assessed visually on a scale from 0 to 5 [29]. Fulton’s condition factor was calculated using the fish fork length [31,32].

Fulton’s condition factor was calculated as follows:

K = 100 · W · FL^−3^

where K is Fulton’s condition factor, W is the whole-body wet weight in grams, and FL is the fork length in cm; a factor of 100 is used to bring K close to unity [32].

The age of the fish was determined by examining the scales and vertebrae [33,34]. The warm water immersion method was used to obtain the fish scale specimens [35]. The scales (five to seven from each individual) were soaked in warm water (50–60 °C) for 24 h, and then the scales were cleaned, dried, and mounted between two microscope slides. The labeled mounted scales were examined for annual rings using stereo-microscope MBS-10 “Lomo”. Abdominal vertebrae were cleaned and dried at room temperature. Both scales and vertebrae from the same individuals were examined independently by three operators. Previously published data on the growth and age of the rainbow trout in water bodies of the Balkhash basin (Sidorova [15] and Biryukov [16]) were used for comparison with contemporary data. Two operators learned age identification under the auspices of authors who published the first data on the age of the rainbow trout introduced to the Balkhash basin (Sidorova [15] and Biryukov [16]), greatly lowering possible bias in terms of age estimation between these data and those retrieved from the literature.

The stomach content of 100 individuals from three sites sampled by active fishing gears was preserved in a 10% formalin solution, and then studied in a laboratory using microscopes MBC-10 “Lomo” (St. Petersburg, Russia) (2 × 4, 2 × 7, 4 × 10, 7 × 45) [36,37,38,39]. When possible, aquatic invertebrates were identified to the genera level, and the remains of terrestrial invertebrates to the order level. The frequency of the occurrence of each food item (presence–absence method) was estimated for diet description [37,40].

The acquisition of the fish images was carried out under natural daylight conditions (Camera Panasonic Lumix FZ45, Panasonic, Osaka, Japan). Coloration analysis was performed via automatic fish-to-background segmentation (via the maximization of the class variance in chromatic color space) and estimation of the hue distribution across the whole fish body to visualize the coloration differences using FISCEAPP: Fish Skin Color Evaluation Application. For this, standardized light conditions and white balance correction on the camera are expected. Finally, images of six rainbow trout from the Masak brooks and ten specimens each from the Ulken Kokpak River and Lower Kolsay Lake were used. The fish body in each image was the dominant object and the background was semi-uniform. Then, Expertomica Fishgui (a standalone Matlab application) was used to evaluate skin coloration in the RGB, HSV, and CIE L*a*b* color spaces, plus the value of the dominant wavelength (lambda). As a result of the processing, two graphs were plotted: the position of the average pixel in the chromaticity diagram and the non-normalized color distributions across the images in the RGB color space [41,42].

### 2.3. Descriptive Statistics

Standard univariate analysis methods were used to establish the general characteristics of the samples from each population [43]. Pairwise comparisons of length, weight, and condition factor were carried out using the Mann–Whitney test (U). Additionally, the paired T-test was used to compare fish common growth in different rivers (T). The correlation between the abiotic parameters of water and fish species, as well as the pairwise presence/absence of fish species, was calculated from the correlation matrix using the Spearman coefficient. Spearman’s correlation coefficients were considered significant at the 0.5 level and 95% confidence level (*p* < 0.05). We also compared the samples by fish species composition using canonical correspondence analysis (CCA) [44] in the PAST software. CCA is a method that extracts the best synthetic gradients from field data on biological communities and environmental features.

## 3. Results

### 3.1. Modern Distribution and Co-Occurrence with Other Fish Species

**Figure 2:**
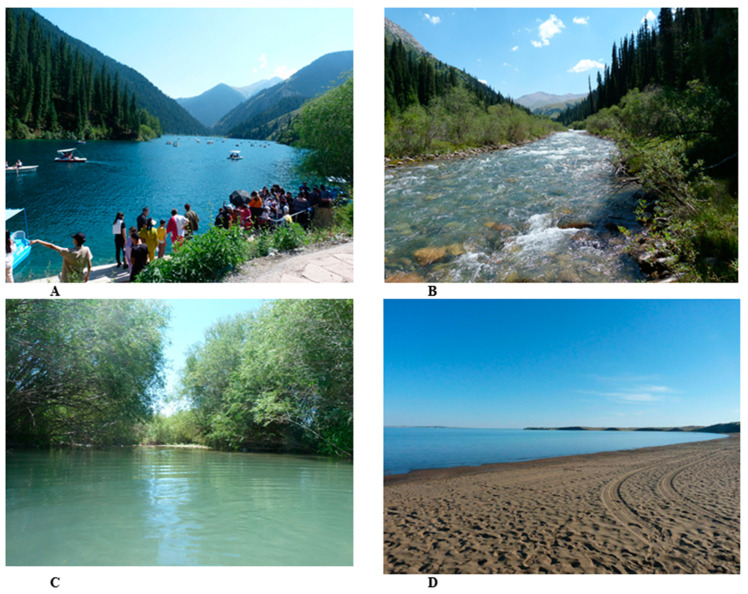
Water bodies inhabited by *O. mykiss*: (**A**) Lower Kolsay Lake; (**B**) the Ulken Kokpak River; (**C**) a branch of the Chilik River near Masak; (**D**) Kapchagay Reservoir.

*O. mykiss* was recorded in ten localities in basins of the Ili River and was found to be absent in other water bodies of the Balkhash basin (Figure 1). The habitats in the Ili River watershed might be subdivided into four types: (a) high-mountain lakes, represented by Lower and Middle Kolsay Lakes (1872 and 2259 m a.s.l., respectively); (b) high-mountain fast-running channeled rivers, represented by the Ulken Kokpak (2106–2300 m a.s.l.), Shalkudysu (2100–2250 m a.s.l.), Zhinishke (1300–1650 m a.s.l.), Issyk (1300–1760 m a.s.l.) and Turgen (1100–1800 m a.s.l.) rivers; (c) lowland springs, brooks, river branches, and channels, represented by Masak brooks (591 m a.s.l.) and the Babatogan branch of the Chilik River (492 m a.s.l.); and (d) the Kapchagay Reservoir (481 m a.s.l.) (Figure 2). Kolsay Lakes and Ulken Kokpak River are geographically remote water bodies. The Issyk and Turgen rivers and Lower Kolsay Lake have recently become very popular places for recreation and recreational fishing (see Figure 2A). On summer days, up to 10 fishers per kilometer of river length were observed on the Turgen and Issyk rivers. All fishermen used their catch for food. Unfortunately, illegal fishing methods were also observed (remains of gill nets; empty bags of bleach) in the Lower Kolsay Lake and in the upper reaches of the Turgen River. Recently, the Zhinishke and Ulken Kokpak rivers have also attracted the attention of game fishermen.

The rainbow trout inhabited localities with different water characteristics (Table 1). Some of them had quite high temperatures and water turbidity in summer. Nevertheless, these fish have typically existed here for a long time. Several dams for irrigation purposes were constructed on the middle and lower sections of the Tekes, Charyn, and Chilik rivers. None of these structures feature fish passages; thus, fish migration is only possible unidirectionally from the upper to the lower section. Rainbow trout have not been found in the Tentek and Emel rivers.

The rainbow trout co-occurs with many other fish species inhabiting the different water bodies of the Balkhash basin (Table 2; common names of the fish species are given in Table S3). The rainbow trout was the dominant species in Lower Kolsay Lake. Native scaly osman *Diptychus maculates* Steindachner, 1866 inhabits the headwaters of the Ulken Kokpak River, while naked osman *Gymnodiptychus dybowskii* (Kessler, 1874) and Tibetan stone loach *Triplophysa stolickai* (Steindachner, 1866) inhabit the headwaters of the Turgen and Issyk rivers. Downstream, the rainbow trout appears and native species disappear. Native fish species also dominated the mountain Shalkudysu River and lowland Masak brooks. These three rivers are rather short, and appropriate sites for rainbow trout are limited. In Kapchagay Reservoir, the rainbow trout were recorded as co-occurring with other alien species. In this study, this species was not recorded in the basin of Alakol Lakes.

**Figure 3:**
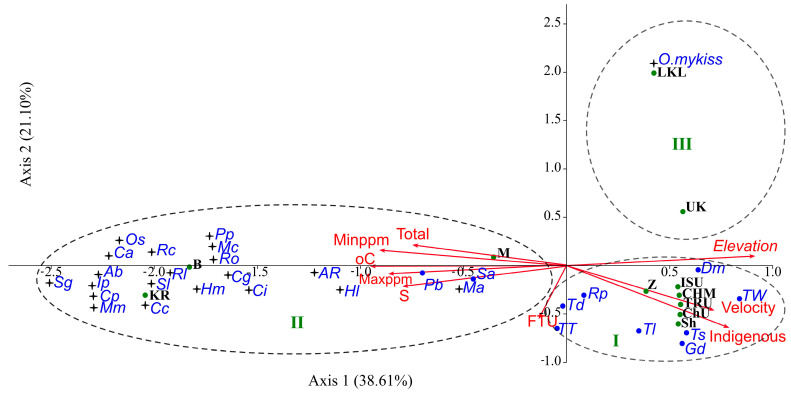
CCA biplot of fish species and environmental variables for rainbow trout. Arrows show species distributions along gradients of the variables. Abbreviations of variables (red), fish names (blue) and water bodies (green) are shown. Variables: Total—common fish abundance; Minppm—minimal concentration of ions; Maxppm—maximal concentration of ions; °C—water temperature; FTU—water turbidity; Velocity—water velocity; Elevation—elevation of the sites above sea level. Water bodies: LKL—Lower Kolsay Lake, UK—the Ulken Kokpak River, ISU—the Issyk River (upper), CHM—the Chilik River (middle reach), TRU—the Turgen River (upper), ChU—the Charyn River (upper), Sh—the Shalkudysu River, M—Masak springs and brooks, B—Babatogan, KR– the Kapchagay Reservoir. Fishes: *O. mykiss*—*the rainbow trout*; *Dm*—*D. maculatus*; *Gd*—*G. dybowskii*; *Sa*—*S. argentatus*; *Pb*—*P. brachyurus*; *Rp*—*R. poljakowi*; *Ts*—*T. stolickai*; *Td*—*T. dorsalis*; *TT*—*T. strauchii*; *Tl*—*T. labiata*; *TW*—*T. sewerzowii; Cp*—*C. peled*; *Cg*—*C. gibelio*; *Cc*—*C. carpio*; *Ab*—*A. brama*; *Rl*—*R. lacustris*; *Ci*—*C. idella*; *Hm*—*H. molitrix*; *Mm*—*M. mantschuricus*; *AR*—*A. rivularis*; *Pp*—*P. parva*; *Hl*—*H. leucisculus*; *Ro*—*R. ocellatus*; *Ma– M. anguillocaudatus*; *Sg*—*S. glanis*; *Ip*—*I. punctatus*; *Ol*—*O. sinensis*; *Sl*—*S. lucioperca*; *On*—*O. niloticus*; *Mc*—*M. cintus*; *Rc*—*R. cheni*; *Ca*—*C. argus.* Cross marks indicate alien species, and blue dots indicate native ones.

The rainbow trout is able to coexist with most fish species inhabiting different water bodies of the Balkhash basin (Table 2). CCA analysis of fish species composition showed the subdivision of water bodies into three groups (Figure 3): (i) water bodies of the piedmont zone mainly inhabited by native species; (ii) the plain zone of the Chilik River and Kapchagay Reservoir mainly inhabited by alien species; and (iii) Lower Kolsay Mountain Lakes and the Ulken Kokpak River mainly inhabited by the rainbow trout, among other species.

### 3.2. Life History Traits

**Figure 4:**
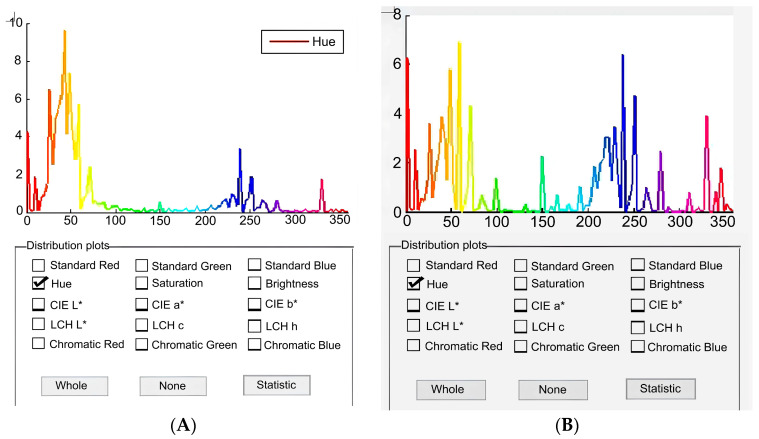
Results of the rainbow trout skin coloration analysis: (**A**) fishes from Masak; (**B**) fishes from the Ulken Kokpak River. Differences are shown in the amount of blue hue.

The common appearance of all investigated fishes corresponds to previous descriptions [2,3,4]. One difference in body coloration was revealed between fish from mountain water bodies and lowland Masak brooks. The skin of all investigated fishes from the Masak tends to be clearly orange-hued (Figure 4). All fishes from the Ulken Kokpak River and Lower Kolsay Lake also have a clear orange peak, as well as a strong blue peak. The strength of the peak differs according to the fish, but the hue position is always consistent, and the profiles are clearly distinct.

The general characteristics of the investigated samples are presented in Table 3. Despite obvious differences in habitat, rainbow trout from Lower Kolsay Lake and the Ulken Kokpak River are characterized by similar size, weight, condition, and fatness (U, *p* < 0.01). The characteristics of samples from the Shalkudysu River (cold mountain river) and the Babatogan branch of the Chilik River (warm lowland river) are within the same range of variation (U, *p* < 0.01). In the Masak brooks (lower section of the Chilik River) and the Zhinishke River (mountain river), the fish were smaller in body size than samples from the Ulken Kokpak River and Lower Kolsay Lake (U, *p* < 0.01). The fatness coefficient of samples from mountain water bodies varies over a wide range. There was no significant correlation (*p* > 0.05) between the size of the fish and their condition factor or between the age and condition factors. Many individuals of the rainbow trout in Lower Kolsay Lake did not have mesenteric fat around the pyloric caeca in autumn 2021 and spring 2022, contrary to samples from other localities. Samples from the Masak brooks were the fattest, having a considerable amount of fat (4 or 5 degrees) in the visceral cavity (Figure S2). The population from Lower Kolsay Lake and Ulken Kokpak River had a lower minimal number of pyloric caeca (18) compared to samples from other water bodies (>40 pyloric caeca).

**Figure 5:**
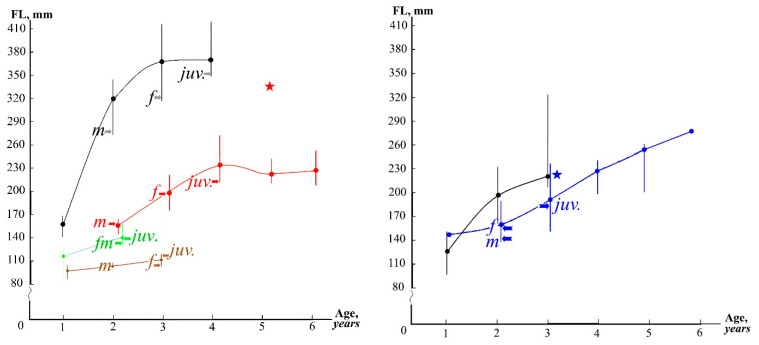
Rainbow trout growth. Left: red line: Lower Kolsay Lake; green line: Masak brooks; brown line: the Zhinishke River; red star: Kapchagay Reservoir; black line: Lower Kolsay pioneers (1966–1969) by [15]. Right: blue line: the Ulken Kokpak River; blue star: the Shalkudysu River; black line: the Uryukty River pioneers (1975–1979) by [16]. Dots show averages, and vertical lines show limits. Letters and arrows show *juv.*, maximum size and age of juveniles; *m* and *f*, minimum length and age of males and females, respectively.

In Lower Kolsay Lake, some individuals (0.14) with an FL of 190 mm were immature while all the fish were sexually mature in the Ulken Kokpak River (Figure 5). In the Zhinishke River, immature fish predominated (0.63), although sexually mature males with a body length of about 90 mm were noted. Mature fish predominated (0.88) in the Masak brooks. Among the mature fish in all water bodies, males were more numerous than females, by about 2.5 times.

Young and adult fish were present in Lower Kolsay Lake, the Ulken Kokpak, Zhinishke rivers, and the Masak brooks, indicating suitable conditions for rainbow trout reproduction and sustainability. Fish from the Lower Kolsay Lake and the Ulken Kokpak River show similar average growth rates and ranges of individual size values of fish of the same age (U and T tests, *p* > 0.05) (Figure 5). Individuals from Zhinishke River grew more slowly (U and T tests, *p* < 0.01).

The composition of the trout diets is given in Table 4. The larvae and imago of amphibiotic insects were the dominant food, and no stomachs contained fish prey despite the co-occurrence of the rainbow trout with other fish species. Amphipods *Gammarus* sp., Trichoptera, and Ephemeroptera larvae are permanent food sources for the rainbow trout during all the seasons. Terrestrial insects are a major food source for the species during the warm season when the present local native fish feed mostly on water invertebrates during warm seasons (Table S4) and demonstrate low feeding activity in cold seasons.

## 4. Discussion

Our results showed successful adaptation of the rainbow trout to lacustrine (Kolsay Lakes) and riverine (basins of the Chilik and Tekes Rivers) environments, as well as mountain (cold water) and lowland (warm water) parts of the Chilik River. Within the Tekes River, this species is distributed in the upper reaches of the Ili River system in the neighboring People’s Republic of China [45]. There are private fish farms in the mountainous areas of the Turgen and Issyk Rivers, where the fish periodically escape to the natural environment. The self-reproductive cultured rainbow trout were totally extirpated from the Turgen River in the early 2000s [21,46]. At present, only triploid rainbow trout from abroad (Denmark, Poland, and Turkey) are cultivated in Kazakhstan [47,48]. These triploid forms of the trout are believed to be sterile [49]. Thus, self-reproducing populations do not exist in the Turgen and Issyk Rivers. No rainbow trout has been reported to originate from the Tentek and Emel Rivers [50,51]. The rainbow trout widely dispersed throughout the whole basin from the sites of their first introduction [6,7,8,9]. Notably, the rainbow trout have not colonized other tributaries of the Ili River system outside rivers of their initial introduction. For instance, the Borokhudzir and Usek Rivers are hydrologically connected to the water bodies where *O. mykiss* was first introduced and offer rather suitable environments based on the investigated water parameters and fish assemblages. However, these tributaries were not populated by the rainbow trout.

*O. mykiss* belongs to salmon species with well-pronounced homing; intraspecific competition manifests itself in the diversity of lifestyles and is one of the incentives for the development of new habitats [25,26,27,28].

The differences in the age and size of maturation in populations from Lower Kolsay Lake and the Ulken Kokpak River demonstrated the high plasticity of their life history traits (Figure 5). Intrapopulational divergences were noted during the years following their introduction [15,16]. The growth rate of contemporary populations of the rainbow trout from Lower Kolsay Lake has noticeably decreased compared to that noted in the fish first introduced [15]. Contrary, the maximal life span in the naturalized population found in Kolsay Lakes (introduced from European farms) has increased from 6 years [15] to up to 10 years old, as seen in fish caught in Middle Kolsay and Lower Kolsay Lakes [19,20]. In the Ulken Kokpak River, fish older than 3 years were not found previously [16], and 4–6-year-old individuals are rarely recorded currently.

Remarkably, there are many spots with cold springs in the Masak brooks that do not exceed 14 °C in summer, but all the rainbow trout were recorded in water with temperatures of ≥19 °C. Taking this observation, we cannot state that rainbow trout prefer cold refugia. Records of rainbow trout in warm waters are rare and can be explained by the presence of either temperature shelters due to depth or cold springs [23,52] or physiological adaptations [53,54,55]. The rainbow trout from the warm waters of the Masak region had a significant amount (4 or 5 grade) of mesenteric fat (Figure S2), possessing a growth rate comparable to that of other such populations, like those found in the cold water of the Zhinishke River. This contradicts the previous observations [56] of less fat and lower linear growth in fish inhabiting warm riverine segments. Our data show that age, rather than size or condition factors, is possibly more important in terms of influencing the sexual maturation of rainbow trout in warm waters. The other important parameter is the different hue of the skin of the rainbow trout from the Mask brooks; Colihueque [57] pointed out the genetic determination of skin hues of rainbow trout. DNA analysis of the rainbow trout is required in future studies focusing on the Balkhash basin.

Data on stomach contents, condition factors, and mesenteric fat suggest the existence of a sufficient food base for the rainbow trout in the water bodies of the Balkhash basin. All of the examined fish fed exclusively on water and terrestrial insects. Surprisingly, none of the hundred investigated fish were found to be predating other fish. Some dietary changes have occurred between their first introduction (1960–1970s) and in contemporary populations. The pioneering population of the rainbow trout in Lake Lower Kolsay predated on native fishes—loaches *Triplophysa* sp. and the naked Osman *Gymnodiptychus dybowskii*. However, after a decline in prey populations, this species changed its diet, consuming invertebrates only [58]. In the first years following their introduction, the rainbow trout from Kamchatka consumed mainly aquatic invertebrates [16]; currently, however, flying insects, especially flies and beetles, frequently appear as food sources for populations found in the Ulken Kokpak River. One may suggest that the non-piscivorous feeding mode of the naturalized rainbow trout can be explained by the low number of pyloric caeca. An experimental study [59] showed that fish with more pyloric caeca assimilated such food better than those with fewer pyloric caeca. Two non-exclusive hypotheses could account for the relationship between growth and caeca number: the number of caeca could be either an indicative characteristic that reflects initial growing conditions or a causal characteristic acting on intestinal morphology and food utilization [59].

Despite the great similarity revealed in terms of the biological needs between the rainbow trout and naked osman, scaly osman, and Tibetan stone loach [60,61,62] (Table S4), the native fishes have successfully coexisted for decades with the predators. The naturalization of the rainbow trout in the water bodies of the Balkash basin is a rare case [63,64,65] in that this species has not caused significant damage to the present native species. However, its relationships with local fauna have not yet been sufficiently studied to draw final conclusions.

**Figure 6:**
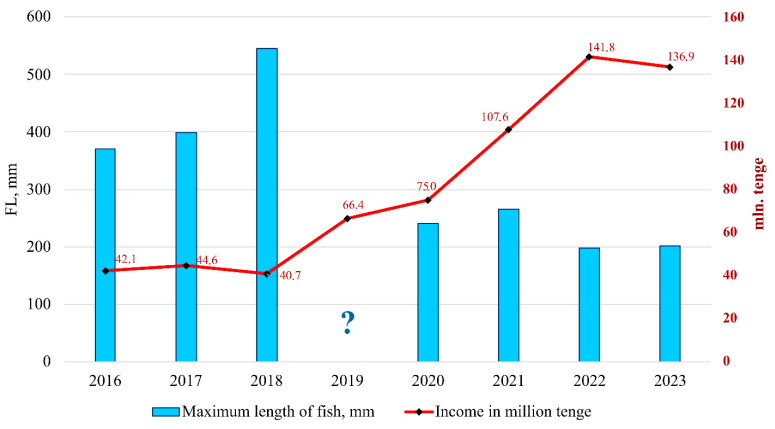
Correlation of maximum body length (FL) of rainbow trout in Lower Kolsay Lake with income (in national currency: KZT) of the “Kolsay Koldery” National Nature Park, showing the impact of recreational fishing.

All of the studied populations of the rainbow trout in the Balkhash basin are subject to intensive recreational fishing. In addition, the population in Kolsay Lakes is under pressure caused by illegal fishing [19,20]. The selective catching of large-sized (and probably rapidly growing) individuals has decreased the body size of old-aged fish in these lakes (Figure 5). Based on data concerning the income of the “Kolsay Koldery” National Nature Park [66], the rapid increase in visits began in 2020. This strongly correlates with the decreases in the maximum body length of the rainbow trout in Kolsay Lakes (correlation r = −0.820, Figure 6).

Our data coincide with those of a previous study [67], which revealed that intensive angling pressure on an invasive fish species (e.g., pumpkinseed *Lepomis gibbosus*) results in a smaller size at maturity and an overall slower-growing phenotype.

## 5. Conclusions

1. Both the wild rainbow trout from Kamchatka and the cultured rainbow trout from Europe are well adapted to the Balkhash basin. Despite the revealed diversity of lifestyles reflecting intrapopulation competition, this species has not spread beyond the river basins of its initial release after 50 years of naturalization.
2. The observed differences between individual rainbow trout in terms of their growth rate, size, weight, and maturation age suggest high plasticity and different life strategies realized within these populations, even in small water bodies. The rainbow trout from the lowland differ in body color from the fish found in mountainous areas.
3. The rainbow trout naturalized in the water bodies of the Balkhash system are characterized by a non-piscivorous feeding mode. The rainbow trout is not an evident threat to the present local native fish.
4. The self-reproducing populations of the rainbow trout naturalized to the environment of the Balkhash water bodies might be an important source for the development of local strains for aquaculture and food security in the Republic of Kazakhstan. However, this species is a facultative piscivore in its natural range, as well as in many novel invasive ranges [38,68,69,70]. Therefore, the monitoring of this alien fish species is extremely necessary to preserve native fish fauna and ensure ecosystem welfare.

## Figures and Tables

**Table 1:** Water characteristics of studied localities during summer (minimal–maximal). Maximal values for localities with the presence of rainbow trout are given in **bold**; * 1 m depth.

Sampling Localities	No of Water Bodies in Figure 1	Symbol	°C	pH	ppm	FTU
Rainbow trout present						
Issyk River (upper)	1	ISU	13.4–14.2	7.6–8.0	44–102	0.4–12.3
Turgen River (upper)	2	TRU	14.2–15.3	7.0–7.9	98–124	0.4–17.8
Lower Kolsay Lake	3	LKL	16.2–17.1 *	7.6–8.3	96–128	0.5–2.4
Chilik River (middle reach)	4	CHM	14.3–16.2	7.1–8.2	42–105	3.6–**31.2**
Zhinishke River	4	Z	12.4–16.1	6.7–7.9	38–126	1.7–11.5
Masak springs and brooks	5	M	19.1–24.3	6.5–7.4	234–456	0.5–17.6
Babatogan branch (mouth of the Chilik)	5	B	22.4–**27.3**	6.4–7.6	246–307	1.8–12.4
Kapchagay Reservoir	6	KR	21.0–26.8 *	6.7–8.2	482–**583**	6.3–15.2
Shalkudysu River	7	Sh	12.4–17.4	6.3–7.8	128–134	0.4–5.3
Ulken Kokpak	8	UK	11.5–12.7	7.2–**8.4**	146–155	0.4–3.9
Charyn (upper)	9	ChU	12.1–14.8	6.7–7.9	54–168	0.5–8.1
Rainbow trout absent						
Issyk River (lower)	1	ISL	18.2–26.2	6.3–8.1	257–362	1.1–104
Turgen River (lower)	2	TRL	16.4–25.8	6.5–7.9	384–654	0.5–126
Charyn (lower)	9	ChL	16.3–32.5	6.7–8.8	340–461	0.8–74.6
Ili	10	Ili	18.2–31.5	6.7–8.2	360–777	2.6–103.0
Borokhudzir River	11	Brk	14.2–15.8	6.7–7.5	165–220	0.5–10.2
Usek River	12	Us	12.6–17.3	6.8–7.9	168–192	0.5–16.1
Bolshaya Almatinka (upper)	13	BA	14.2–16.5	6.7–8.2	154–256	0.4–18.0
Shynzhyly	15	Shy	14.2–26.4	7.8–8.3	24–96	1.2–28.3
Tentek River	16	Tn	13.5–26.7	7.1–8.4	24–268	2.6–55.3
Emel River	17	Em	27.8–31.5	7.6–8.3	568–664	2.5–37.4

**Table 2:** Fish species composition in water bodies with alien rainbow trout estimated by total number of fishes for 2020–2023 (as a fraction of one).

Species	Water Bodies (Symbols According to Table 1)										
	ISU	TRU	LKL	CHM	Z	M	B	KR	Sh	UK	ChU
**Alien species**											
Order Salmoniformes											
*Oncorhynchus mykiss*	0.133	0.100	**0.990**	0.047	0.128	0.153	0.007	0.001	0.032	0.323	0.036
*Coregonus peled*	0	0	0	0	0	0	0	0.009	0	0	0
Order Cypriniformes											
*Carassius gibelio*	0	0	0	0	0	0.035	0.071	0.026	0	0	0
*Cyprinus carpio*	0	0	0	0	0	0.012	0.028	0.081	0	0	0
*Abramis brama*	0	0	0	0	0	0.035	0.085	0.391	0	0	0
*Rutilus lacustris*	0	0	0	0	0	0.071	0.277	0.274	0	0	0
*Ctenopharyngodon idella*	0	0	0	0	0	0.024	0.014	0.030	0	0	0
*Hypophthalmichthys molitrix*	0	0	0	0	0	0.006	0.007	0.012	0	0	0
*Megalobrama mantschuricus*	0	0	0	0	0	0	0	0.005	0	0	0
*Abbottina rivularis*	0	0	0	0	0	0.012	0.007	0.002	0	0	0
*Pseudorasbora parva*	0	0	0	0	0	0.018	0.071	0.005	0	0	0
*Hemiculter leucisculus*	0	0	0	0	0	0.012	0.007	0	0	0	0
*Rhodeus ocellatus*	0	0	0	0	0	0.018	0.085	0	0	0	0
*Misgurnus anguillocaudatus*	0	0	0	0	0	0.024	0	0	0	0	0
Order Siluriformes											
*Silurus glanis*	0	0	0	0	0	0	0	0.045	0	0	0
*Ictalurus punctatus*	0	0	0	0	0	0	0	0.005	0	0	0
Order Beloniformes											
*Oryzias sinensis*	0	0	0	0	0	0	0.184		0	0	0
Order Perciformes											
*Sander lucioperca*	0	0	0	0	0	0.012	0.035	0.104	0	0	0
*Micropercops cintus*	0	0	0	0	0	0.006	0.028	0	0	0	0
Order Cichliformes											
*Oreochromis niloticus*	0	0	0	0	0	0	0	0	0	0	0
Order Gobiiformes											
*Rhinogobius cheni*	0	0	0	0	0	0	0.021	0	0	0	0
Order Anabantiformes											
*Channa argus*	0	0	0	0	0	0	0.035	0.011	0	0	0
**Indigenous species**											
Order Cypriniformes											
*Diptychus maculatus*	0	0	0	0.186	0.048	0	0	0	0.258	0.548	0.214
*Gymnodiptychus dybowskii*	0.600	0.825	0.01	0.380	0.432	0.188	0	0	0.581	0	0.536
*Schizothorax argentatus*	0	0	0	0	0	0.071	0	0	0	0	0
*Phoxinus brachyurus*	0	0	0	0	0	0.047	0.007	0	0	0	0
*Rhynchocypris poljakowi*	0	0	0	0.008	0	0.024	0	0	0	0	0.012
*Triplophysa stolickai*	0.178	0.025	0	0.380	0.176	0.024	0	0	0.129	0.129	0.167
*Triplophysa dorsalis*	0	0.050	0	0		0.047	0.007	0	0	0	0
*Triplophysa strauchii*	0	0	0.01	0	0.216	0.147	0.021	0	0	0	0
*Triplophysa labiata*	0.089	0	0	0	0	0.018	0	0	0	0	0
*Triplophysa sewerzowii*	0	0	0	0	0	0	0	0	0	0	0.036
**Total number of fishes**	45	40	200	129	125	170	141	939	31	62	84
**Number of indigenous species**	3	3	2	4	4	8	3	0	3	2	5
**Number of alien species**	1	1	1	1	1	13	16	15	1	1	1
**Sampling time, hours**	34	52	67	39	36	74	30	76	33	36	34

Origin of the fishes, the common characteristics of the samples and the maximal observed proportion of the rainbow trout given by bold.

**Table 3:** Body size (SL); weight (Q); Fulton’s condition factor (K); and quantity of mesenteric fat (Fat). Above line: min—minimal and max—maximal; below line: M—average and ±SD—standard deviation; n—sample size.

Water Body and Sample Size	SL (mm)	Q (g)	K	Mesenteric Fat
	min–max M ± SD	min–max M ± SD	min–max M ± SD	min–max M ± SD
Lower Kolsay, n = 20	127–237 176.2 ± 28.79	45.0–238.0 112.0 ± 49.70	1.06–1.51 1.34 ± 0.125	0–2 1.1 ± 0.94
Ulken Kokpak, n = 20	125–318 178.1 ± 48.20	34.0–546.1 141.3 ± 128.89	1.22–1.72 1.39 ± 0.127	0–3 1.1 ± 1.02
Masak and Lower Chilik, n = 26	82–156 113.0 ± 13.93	5.8–56.1 31.1 ± 13.60	1.13–1.71 1.44 ± 0.140	4–5 4.5 ± 0.52
Zhinishke, n = 16	78–109 89.9 ± 6.57	9.7–22.1 15.2 ± 3.50	1.28–1.48 1.40 ± 0.059	1–4 2.5 ± 0.71
Shalkudysu, n = 1	193	120.0	1.13	2
Babatogan, n = 1	185	145.7	1.62	4
Kapchagay Reservoir, n = 1	296	564.0	1.54	2

**Table 4:** Diet composition of the rainbow trout in the water bodies of Balkhash basin. Months are indicated by Roman numerals; n, sample size.

Food Items	Lower Kolsay Lake			Ulken Kokpak River			Masak Brooks		
	IV	VI	IX	V	VI	IX	III	VIII	IX
	n = 16	n = 20	n = 18	n = 6	n = 15	n = 5	n = 3	n = 12	n = 5
Aquatic invertebrates									
Annelida: Hirudinea: Rhynchobdellidae	0.063	0.100	0	0.167	0.067	0	0	0.250	0.200
Mollusca: Bivalvia: Sphaeriida: *Pisidium amnicum*	0.188	0	0.222	0	0	0	0	0	0
Arthropoda: Crustacea:									
Cladocera	0.063	0.400	0	0	0.133	0	0	0.583	0
*Gammarus* sp.	0.688	0.750	0.778	0.833	0.267	0.200	1.000	0.833	0.400
Insect larvae and pupae:									
Trichoptera	1.000	0.850	1.000	1.000	1.000	1.000	0.333	0.250	0.400
Ephemeroptera	1.000	0.600	0.944	0.833	0.800	1.000	1.000	0.917	1.000
Plecoptera	0.563	0.300	0.833	1.000	0.733	0.800	0.667	0.333	0.600
Odonata	0	0.150	0	0.167	0.333	0.200	0.667	0.583	0.200
Diptera	1.000	1.000	1.000	1.000	1.000	1.000	1.000	1.000	1.000
Terrestrial inveretbrates; insect adults (imago):									
Trichoptera	0	0.600	0	0	0.267	0	0	0.167	0
Plecoptera	0	0.250	0	0	0.400	0	0	0.167	0
Hymenoptera	0.188	0.650	0.056	0	0.733	0.600	0	0.583	0.800
Hemiptera	0.063	0.350	0	0.167	0.133	0	0	0.083	0
Orthoptera	0	0.400	0	0.333	0.333	0.200	0	0.583	0.200
Coleoptera	0	0.650	0.111	0.667	0.733	0.600	0	0.750	0.600
Diptera	0	1.000	0.111	1.000	1.000	0.400	0	0.583	0.400
Non-identified food particles	0	0.400	0.389	0.333	0.600	0.600	1.000	0.917	1.000

## Data Availability

The original contributions presented in the study are included in the article/Supplementary Materials; further inquiries can be directed to the corresponding author.

## Supplementary Materials

The following supporting information can be downloaded at https://www.mdpi.com/article/10.3390/ani14203013/s1: Figure S1. Measurements of *Oncorhynchus mykiss*; Figure S2. Mesenteric fat in the rainbow trout from the Masak brooks; Table S1. History of colonization of *O. mykiss* in water bodies of Balkhash basin; Table S2. General characteristics of investigated water bodies and number of *O. mykiss* specimens; Table S3. Valid and common fish names; Table S4. Basic requirements of the rainbow trout and native fish. References [2,4,14,15,16,60,61,62,71,72] are cited in the Supplementary Materials file.
