# Supplementary material for: Alien Rainbow Trout Oncorhynchus mykiss in the Balkhash Basin (Kazakhstan, Central Asia): 50 Years of Naturalization
Source: Animals (Basel). 2024 Oct 18;14(20):3013. doi: 10.3390/ani14203013 (PMC11504185; doi:10.3390/ani14203013)
Supplement: Supplementary file 1 [file animals-14-03013-s001.zip › animals-3127130-supplementary.pdf]

Supplements

Table S1- History of colonization of *O. mykiss* in water bodies of Balkhash basin [14-16]

Originated from	Introduced in	Year	Realized by (x 1000 specimens)
**Basin of Balkhash Lake**			
Czechoslovakia	Fish farm on Chilik River	1964	200.0 fertilized eggs
		1965	200.0 fertilized eggs
		1966	200.0 fertilized eggs
Czechoslovakia	River Kolsay (Chilik watershed)	1965	2.0 young fish
Czechoslovakia	Lower Kolsay Lake (Chilik watershed)	1965	3.0 young fish
Russia (Fish Selection and Genetic Center, Ropsha)	Lower Kolsay Lake	1969	5.0 young fish
Russia (Fish Selection and Genetic Center, Ropsha)	Middle Kolsay Lake (Chilik watershed)	1969	5.0 young fish
Czechoslovakia	Kaindy Lake (Chilik watershed)	1970	5.0 young fish
Czechoslovakia	Kapchagay reservoir (on Ili River)	1971, 1974	about 2000.0 young fish
Kishimshina River, Kamchatka	Ulken Kokpak River (inflow of Tekes River)	1975	3.0 young fish
Baran'ya River, Kamchatka	Ulken Uryukty Lake (Chilik River watershed)	1978	2.0 young fish
Baran'ya River, Kamchatka	Buzumbay Lake (Tekes watershed)	1978	3.0 young fish
**Basin of Alakol Lakes**			
Kanycheva and Amchigach Rivers, Kamchatka	Tentek River	1976	3.5 young fish
	Emel River	1976	4.0 young fish

Table S2- General characteristics of investigated water bodies and number of *O. mykiss* specimens.

Water bodies	No	Length of rivers or surface area of lakes	Coordinates of sampling localities		Altitude above sea level, m	Number of observed/investigated specimens
			N	E		
Issyk River (upper)	1	96 km	43°15′35″	77°29′06″	1660	6/0
Issyk River (lower)			43°34′25″	77°20′24″	560	0
Turgen River (upper)	2	90 km	43°19′08″	77°38′21″	1715	4/0
Turgen River (lower)			43°34′13″	77°32′34″	582	0
Assy River (top inflow of Turgen River)		67 km	43°15′01″	77°52′00″	2348	0
Lower Kolsay Lake	3	0.352 km^2	42°59′23″	78°19′31″	1822	198/20
Middle Kolsay Lake		0.375 km^2	42°56′14″	78°19′33″	2265	13/0
Kaindy Lake		0.043 km^2	42°59′05″	78°27′52″	1885	2/0
Zhinishke River (top inflow of Chilik)	4	63 km	43°13′41″	78°15′44″	1498	16/16
Chilik (middle reach)		175 km	43°04′05″	78°21′07″	1495	6/0
Masak springs and brooks (low inflows of Chilik)	5	Land area about 30 km^2	43°37′43″	78°17′28″	591	26/26
Babatogan (branch of Chilik)		8 km	43°43′54″	78°10′04″	492	1/1
Kapchagay Reservoir	6	1847 km^2	43°49′36″	78°00′47″	481	1/1
Shalkudysu River (upper inflow of Tekes River)	7	55 km	43°10′33″	79°52′29″	2172	1/1
Ulken Kokpak (Kokpak)	8	50 km	42°40′29″	79°52′10″	2106	20/20
Charyn River	9	155 km	43°41'07"	79°23'53"	637	1/0
			43°54'41"	79°26'54"	488	0
			43°49'38"	79°15'39"	487	0
Ili River	10	1439 km	43°45'26"	80°13'55"	526	0
			43°46'00"	80°16'24"	575	0
			44°05'12"	76°59'29"	471	0
			44°28'52"	76°40'43"	427	0
Borokhudzir River	11	74 km	44°28'54"	79°35'04"	1869	0
			44°15'46"	79°50'21"	862	0
Usek River	12	164 km	44°28'47"	79°49'51"	1357	0
			44°12'41"	79°55'00"	748	0
Bolshaya Almatinka River	13	96 km	43°07'48"	76°54'20"	1322	0
		125 km	43°10'24"	77°01'18"	1377	0

and Malaya Almatinka River			43°30'45"	77°02'06"	586	0
Kaskelen River	14	177 km	43°04'42"	76°36'28"	1438	0
			43°29'34"	76°50'11"	645	0
Shynzhyly River	15	104 km	45°44'57"	80°31'46"	783	0
			46°12'06"	80°48'42"	361	0
Tentek River (upper)	16	167 km	45°59'27"	80°58'57"	1643	0
Tentek River (lower)			46°21'18"	80°55'24"	393	0
Emel River	17	124 km	46°53′20″	82°35′18″	726	0
			46°32′73″	82°17′18″	452	0

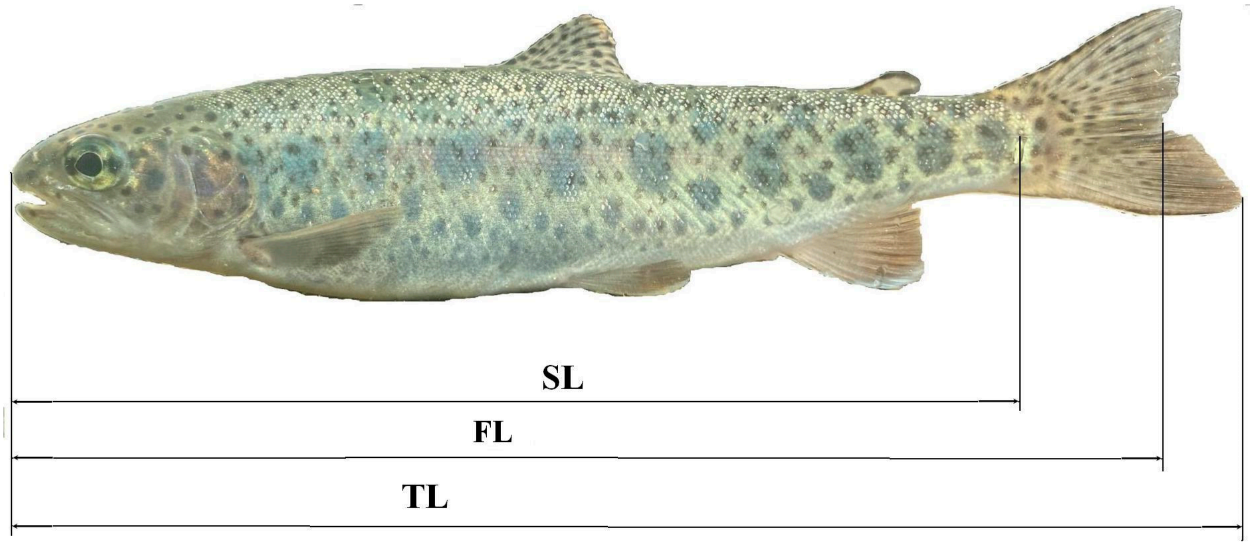

Figure S1 - Measurements of *Oncorhynchus mykiss*: TL – total lehgth, FL – fork length, SL - standard legth

Table S3 - Valid and common fish names

No	Species	Common name
	**Alien**	
**Order Salmoniformes**		
	**Family Salmonidae**	
1	*Oncorhynchus mykiss* (Walbaum, 1792)	Rainbow trout, steelhead trout, or redband trout
	**Family Coregonidae**	
2	*Coregonus peled*	Peled
**Order Cypriniformes**		
	**Family Cyprinidae**	
3	*Carassius gibelio* (Bloch, 1782)	Prussian carp
4	*Cyprinus carpio* Linnaeus, 1758	Carp
	**Family Leuciscidae**	
5	*Abramis brama* (Linnaeus, 1758)	Bream
6	*Rutilus lacustris* (Pallas, 1814)	Roach
	**Family Gobionidae**	

7	*Abbottina rivularis* (Basilewsky, 1855)	Abbottina or false gudgeon
8	*Pseudorasbora parva* (Temminck & Schlegel, 1846)	Topmouth gudgeon
	**Family Xenocyprididae**	
9	*Ctenopharyngodon idela*	Grass carp
10	*Hypophthalmichthys molitrix*	Silver carp
11	*Hemiculter leucisculus* (Basilewsky, 1855)	Korean sharpbelly
12	*Megalobrama mantschuricus*	-
	**Family Acheilognathidae**	
13	*Rhodeus ocellatus* (Kner, 1866)	Rosy bitterling
	**Family Cobitidae**	
	Misgurnus anguillicaudatus	Pond loach
**Order Siluriformes**		
	**Family Siluridae**	
	Silurus glanis Linnaeus, 1758	Wels catfish
	**Family Ictaluridae**	
	Ictalurus punctatus	Channel catfish
**Order Beloniformes**		
	**Family Adrianichthyidae**	
	Oryzias sinensis Chen, Uwa, Chu, 1989	Chinese ricefish
**Order Perciformes**		
	**Family Percidae**	
	Sander lucioperca (Linnaeus, 1758)	Pike-perch
	**Family Odontobutidae**	
	Micropercops cintus (Dabry de Thiersant, 1872)	Beautiful sleeper
**Order Cichliformes**		
	**Family Cichlidae**	
	Oreochromis niloticus	Nile tilapia
**Order Gobiiformes**		
	**Family Gobiidae**	
	Rhinogobius cheni (Nichols, 1931)	Amur goby
**Order Anabantiformes**		
	**Family Channidae**	
	Channa argus (Cantor, 1842)	Snakehead
	**Indigenous**	
**Order Cypriniformes**		
	**Family Cyprinidae**	
	Diptychus maculatus Steindachner, 1866	Scaly osman
	Gymnodiptychus dybowskii (Kessler, 1874)	Naked osman
	Schizothorax argentatus Kessler, 1874	Balkhash marinka
	**Family Leuciscidae**	
	Phoxinus brachyurus Berg, 1912	Seven River’s minnow
	Rhynchocypris poljakowi (Kessler, 1879)	Balkhash minnow
	**Family Nemacheilidae**	
	Triplophysa stolickai (Steindachner, 1866)	Tibetan stone loach
	Triplophysa dorsalis (Kessler, 1872)	Grey stone loach
	Triplophysa strauchii (Kessler, 1874)	Spotted thicklip loach
	Triplophysa labiata (Kessler, 1874)	Plain thicklip loach
	Triplophysa sewrzowii (Nikolsky G., 1938)	Sewertzow’s loach

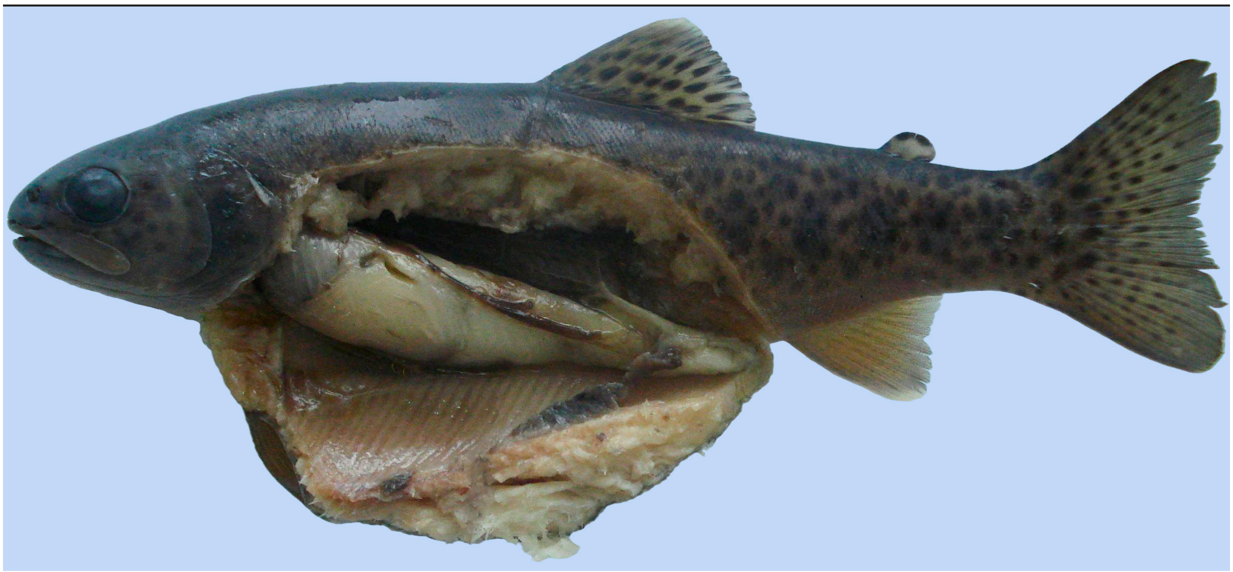

Figure S2 - Mesenteric fat in the rainbow trout from the Masak brooks (SL = 112 mm), degree 5

Table S4. Basic requirements of the rainbow trout and native fish

Fish species	Living conditions			Reproduction traits			
	Preferable biotopes	Optimal temperature (°C)	Preferred feed of adult fish	Age of maturation	Times per year	Fecundity (thousand eggs)	Substrate
Oncorchynchus mykiss [2,4]	Fast-flowing streams, cold lakes	12-21	Benthos and terrestrial insects, fishes	1 year (in Ulken Kokpak) 2-3 (others)	1	1.5-2.5	Gravel
Native fish							
Diptychus maculatus [60]	Fast-flowing streams	8-14	Benthos	3-4	2-3	2.5	Stones
Gymnodiptychus dybowskii [60]	Moderate streams	12-20	Benthos	3-4	2-3	2.5-8.8	Stones, gravel
Schizothorax argentatus [61]	Moderate streams	16-24	Plants	3-4	2-3	30.0-70.0	Gravel, sand
Schizothorax pseudaksaiensis [61]	Lakes and ponds	16-24	Plants, benthos, fish	3-5	1-3	Unknown	Gravel, sand
Phoxinus brachyurus [71]	Brooks, small slow-flowing rivers	16-24	Benthos and terrestrial insects	1	1-3	0.1-0.2	unknown
Rhynchocypris poljakowi [71]	Moderate streams	16-24	Unknown	1	1-2	0.2-0.4	unknown
Triplophysa stoliczkai [62]	Fast and moderate flowing streams	14-24	Algae and benthos	2-3	2-3	3.5-10.5	sand
Triplophysa dorsalis [62]	Slow-flowing rivers, ponds	18-26	Benthos	2	2-3	0.5-4.0	Submerged plants
Triplophysa strauchii	Moderate	16-26	Zooplankton,	2-3	2-3	5.0-20.0	Gravel, sand,

[62]	and slow-flowing rivers, lakes		benthos, algae				submerged plants
Triplophysa labiata [62]	Moderate streams	16-22	Benthos	2-3	2-3	3.0-60.0	submerged plants
Triplophysa sewerzowii [62]	Small slow-flowing rivers	18-26	Benthos	1	2-3	0.2-2.5	submerged plants
Perca schrenkii [72]	Slow-flowing rivers, lakes, ponds	14-22	Benthos, fish	2-3	1	20 -50	submerged plants, stones
